# Structure and function of a *Clostridium difficile* sortase enzyme

**DOI:** 10.1038/srep09449

**Published:** 2015-03-24

**Authors:** Christopher J. Chambers, April K. Roberts, Clifford C. Shone, K. Ravi Acharya

**Affiliations:** 1 Public Health England, Porton Down, Salisbury SP4 0JG, UK; 2 Department of Biology and Biochemistry, University of Bath, Claverton Down, Bath BA2 7AY, UK

## Abstract

Sortase enzymes are responsible for covalent anchoring of specific proteins to the peptidoglycan of the cell wall of gram-positive bacteria. In some gram-positive bacteria (e.g. *Staphylococcus aureus*), sortases have been found to be essential for pathogenesis and their inhibitors are under development as potential novel therapeutics. Here we provide the first report on the structural characterisation of the *C. difficile* sortase. An active site mutant was crystallised and its structure determined to 2.55 Å by X-ray diffraction to provide structural insight into its catalytic mechanism. In order to elucidate the role of the sortase in the cell wall biogenesis, a *C. difficile* sortase knockout strain was constructed by intron mutagenesis. Characterisation of this mutant led to the discovery that the putative adhesin CD0386 is anchored to the peptidoglycan of *C. difficile* by the sortase SrtB and that an SPKTG peptide motif is involved in the transpeptidation reaction with the *C. difficile* peptidoglycan. In an animal model for *C. difficile* infection, the SrtB mutant caused disease at a similar rate of onset as the wild type strain. In conclusion, our detailed study shows that the SrtB enzyme from *C. difficile* does not play an essential role in pathogenesis.

*Clostridium difficile* is an anaerobic enteric pathogen that has risen to great prominence in recent decades due to its ability to cause deadly antibiotic-associated diarrhoea, particularly in healthcare and elderly residential settings^1^,^2^. Emergence of an antibiotic-resistant epidemic 027/B1/NAP1 strain in the early 2000s led to widespread alarm following worldwide outbreaks with unusually high mortality^3^. *C. difficile* is a normal constituent of the colonic flora in 3–10% of healthy individuals, but disruption of the colonic flora by broad spectrum antibiotics creates an ecological niche which *C. difficile* can exploit and cause disease by secretion of toxins^4^. The two classical *C. difficile* toxins, TcdA and TcdB, enter intestinal epithelial cells by endocytosis, and after gaining access to the cytoplasm via their intrinsic pore-forming activity, they selectively O-glucosylate the threonine 37 residue of RhoA and other Rho family proteins leading to dysfunction of the actin cytoskeleton and disruption of the colonic epithelium^5^,^6^. The third toxin, *Clostridium difficile* transferase (CDT), present in around 6% of strains, is an ADP-ribosyl transferase which ADP-ribosylates actin, again causing cytoskeletal dysfunction and epithelial damage. The spectrum of severity of *C. difficile* infection (CDI) ranges from mild diarrhoea to pseudomembranous colitis, toxic megacolon and death. The relapsing nature of the infection and its antibiotic-associated nature has led to a considerable effort to find novel therapeutics, ranging from immunotherapy^7^,^8^ to novel antibiotics which have reduced impact on the gut flora compared to chemotherapies currently available^9^.

As with other toxigenic bacteria such as *Corynebacterium diphtheriae* and enterohaemorrhagic *Escherichia coli* (EHEC), it is thought that *C. difficile* must adhere to the tissue targeted by its toxins in order to maximise the concentration of toxin at the target cells. Although several suspected *C. difficile* adhesins have been identified, targeting cell matrix proteins such fibronectin^10^ and collagen^11^, knowledge of adhesins in *C. difficile* lags behind that of other species. In many gram positive pathogens, adhesins are anchored to the bacterial surface by sortase enzymes, a class of transpeptidases which catalyse a transpeptidation between a peptide motif and the cross-link precursors of the peptidoglycan. The archetypal sortase is SrtA of *Staphylococcus aureus*, which is responsible for the anchoring of proteins that contain a C-terminal cell wall sorting signal (CWSS) including staphylococcal protein A and is therefore essential for pathogenesis^12^.

The C-terminal peptide signal which facilitates anchoring of proteins to the cell wall is tripartite in nature, consisting of (1) a pentapeptide cell wall sorting motif, LPxTG being the prototype sorting motif in *S. aureus*, (2) a largely hydrophobic region 15–20 amino acids in length and (3) a lysine/arginine rich C-terminus of 5–10 amino acids. Following cleavage of the scissile threonyl-glycyl bond within the pentapeptide motif, an acyl intermediate is formed between the target protein and the sortase. This intermediate is resolved by nucleophilic attack by a crosslinked precursor of the peptidoglycan, resulting in the protein being covalently anchored to the peptidoglycan.

The discovery that SrtA is essential for pathogenesis of *S. aureus* has led to much interest in sortases as novel targets of anti-infective agents, and several sortase inhibitors have been described^13^,^14^. The *C. difficile* 630 genome possesses two genes with homology to known sortase genes in other species, but as one of these is interrupted by an in-frame stop codon, it is thought that only one sortase (SrtB) is present in the bacterium. A recent study demonstrated that SrtB recognises the LPxTG motif within putative sortase substrates^15^. Given that novel therapeutics for *C. difficile* are desperately sought, it was considered imperative to investigate the *C. difficile* sortase to determine whether this enzyme could be a potential target of future therapies. In this report, we describe the crystal structure of *C. difficile* SrtB, biochemical analysis of its reaction products and characterisation of a *C. difficile* mutant deficient in its sortase enzyme.

## Results

### In-silico identification of the putative *C. difficile* sortase substrates

A BLAST search to identify hypothetical *C. difficile* 630 proteins containing an [SP]PxTG motif led to a preliminary list of 25 proteins. Of these, 15 were eliminated because they did not contain an N-terminal secretion signal peptide as determined by the SignalP program. The remaining proteins were examined manually, and a further 3 eliminated either because the [SP]PxTG motif was too far (<50 amino acids) from the C-terminus or because the C-terminus was not rich in positively charged amino acids. The remaining seven proteins, containing all of the characteristics of sortase anchored proteins, are listed in Table 1 accompanied by gene annotations and results of conserved domain searches.

### Expression and crystallisation of *C. difficile* SrtB and SrtB C226A

**Figure 1:**
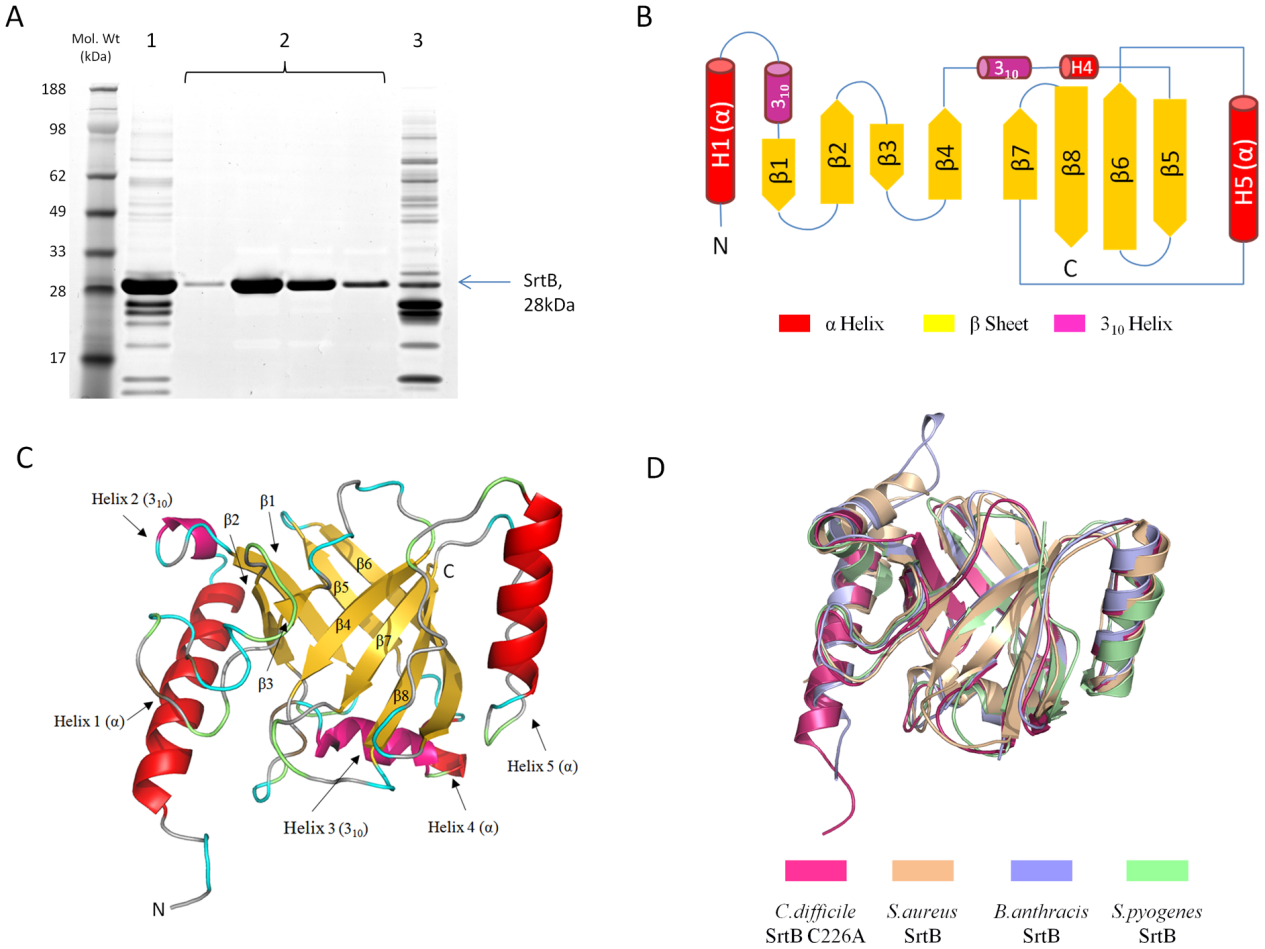
(A) Final purification of recombinant *C. difficile* SrtB C226A mutant. Nickel affinity purified SrtB (1) was applied to a 5 ml Butyl sepharose column in a buffer containing 1 M (NH_4_)_2_SO_4_. SrtB flowed through the column at >95% purity (2) while the majority of contaminants bound to the column and were eluted on application of a low ionic strength buffer (3). (B) Schematic of *C. difficile* SrtB C226A coloured by secondary structure. A schematic of secondary structure elements clearly shows the non-contiguous nature of the barrel from β4 onwards. (C) *C. difficile* SrtB C226A Coloured by secondary structure. The structure of *C. difficile* SrtB incorporates three α-helices, two 3_10_-helices and eight β-strands. (D) Superposition of *C. difficile* SrtB with several class B sortase structures. The *C. difficile* SrtB C226A structure superposed with the class B sortases of *S. aureus* (PDB 1NG5), *B. anthracis* (PDB 1RZ2) *and Streptococcus pyogenes* (PDB 3PSQ).

Two forms of *C. difficile* SrtB were expressed recombinantly – SrtB, consisting of the *C. difficile* 630 open reading frame CD2718 omitting the predicted secretion signal at positions 1–32, and a C226A mutant of this protein based upon the inactive *S. aureus* SrtA mutant described by Ton-That et al.^16^ Expression of *C. difficile* SrtB and SrtB C226A in *E. coli* BL21 yielded a soluble polyhistidine-tagged protein which could be purified to >95% purity by a two step protocol consisting of a nickel affinity column followed by a hydrophobic interaction column which bound the remaining contaminants while allowing SrtB to flow through. SDS-PAGE analysis of purified SrtB is shown in Figure 1A. The molecular weights of SrtB and SrtB C226A were determined by electrospray mass spectroscopy as 28308 and 28276 respectively, confirming a loss in mass of 32Da in SrtB C226A due to mutation from cysteine to alanine. High throughput crystallisation screening with both SrtB and SrtB C226A yielded small crystals in one condition with SrtB C226A, consisting of 0.1 M Phosphate/Citrate buffer pH 4.2, 40% PEG300. Larger crystals were obtained by micro seeding of these small crystals into pre-equilibrated drops containing the same condition but with a slightly lower (32%) precipitant concentration.

### Determination of the *C. difficile* SrtB C226A crystal structure

X-ray diffraction data were collected at beam line I04-1 of the Diamond Light Source (Didcot, Oxon) UK. A summary of data collection and processing statistics are shown in Table 2. A resolution limit of 2.55 Å was applied as the *R*_merge_ above this resolution was considered unacceptably high. Structure determination by molecular replacement with PHASER-MR^17^ was attempted using all available sortase structures in the Protein Data Bank (PDB) at the time of analysis. The lowest translation and rotation Z scores were obtained using the crystal structure of the *Bacillus anthracis* Sortase B (PDB 1RZ2)^18^. A homology model of *C. difficile* SrtB was constructed with SWISS-MODELLER using the structure 1RZ2 (from the Protein Data Bank) as a template, and this model used to solve the crystal structure by molecular replacement with PHASER^17^. Structure refinement was performed iteratively across the whole assembly using REFMAC^19^ for automated refinement and COOT^20^ for real space refinement following inspection of electron density. The final refinement statistics for the *C. difficile* SrtB structure are listed in Table 2.

### Structure of *C. difficile* SrtB C226A

**Figure 2:**
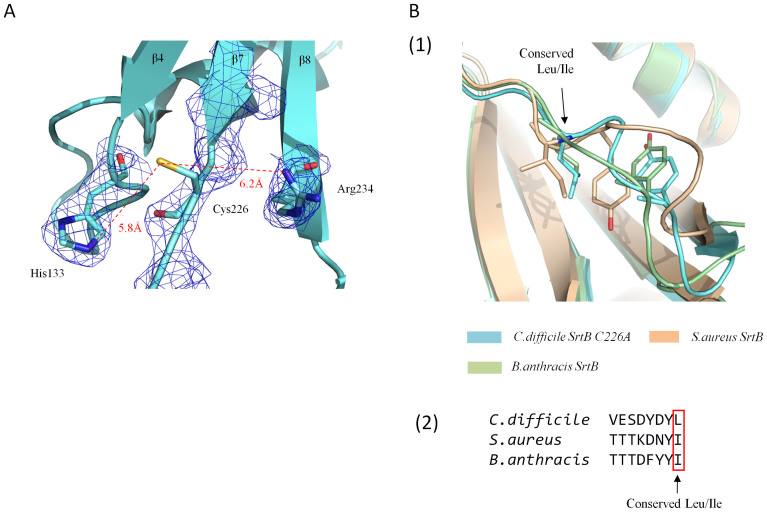
(A) The modelled active site of *C. difficile* SrtB. Portion of the electron density map (blue mesh) is shown contoured to 1.0 Sigma. Clear and continuous electron density is visible for His133 and Arg234. Electron density is clearly absent for the modelled Cys266. (B) The putative CWSS peptide binding loop of three superposed SrtB enzymes. (1) The β6-H5 loop of *C. difficile* SrtB, *S. aureus* SrtB and *B. anthracis* SrtB all contain a conserved Leu/Ile residue and a structurally semi-conserved tyrosine residue. (2) The conserved polar nature of the amino acids preceding the conserved Leu/Ile is clear in the primary sequence of the region.

The arrangement of secondary structure elements and overall fold of *C. difficile* SrtB C226A, shown in Figure 1B and 1C respectively, is typical of a class B sortase. The N-terminus is characterised by a substantial alpha helix of 18 amino acids long followed by a short loop incorporating a three residue 3_10_ helix. Strands β1 and β2 are arranged in an antiparallel manner, separated by a turn motif. A long loop places β3 (which is the shortest of the beta sheet regions) parallel to β2, while β4 is antiparallel to β3. Following β4, another 3_10_ helix of 7 residues is followed immediately by a shorter alpha helix of 4 residues. This breaks the continuity of the barrel by placing β5 adjacent to β1 rather than β4. The longest extended beta sheet region of 13 amino acids, β6, lies antiparallel to β5 for around a third of its length and forms a striking curved backbone of the barrel. The long sequence separating β6 from β7 features an alpha helix of 12 residues in length. The strand β7 runs parallel to β4, with β8 completing the barrel by running between and antiparallel to β6 and β7. No metal ions were observed in the β6/β7 loop known to participate in Ca^2+^ ion binding in *S. aureus* SrtA. This is not surprising given that the loop is devoid of the triad of glutamine residues which co-ordinate metal binding in *S. aureus* SrtA. The modelled active site of *C. difficile* SrtB, found on the edge of the barrel at the base of β7 and shown in Figure 2A, has a disposition similar to that seen in other sortases, consisting of a cysteine residue (C226) slightly beyond the C-terminus of β7 flanked by a histidine residue (H133) at the C-terminus of β4 and an arginine residue (R234) within the N-terminal portion of β8. The cysteine and arginine residues are found in a deep depression on the surface of the enzyme, while His133 is shielded from the depression by the side chain of tyrosine residue 227 within the β7/β8 loop.

### HPLC and LC-MS analysis of recombinant *C. difficile* SrtB reactions

**Figure 3:**
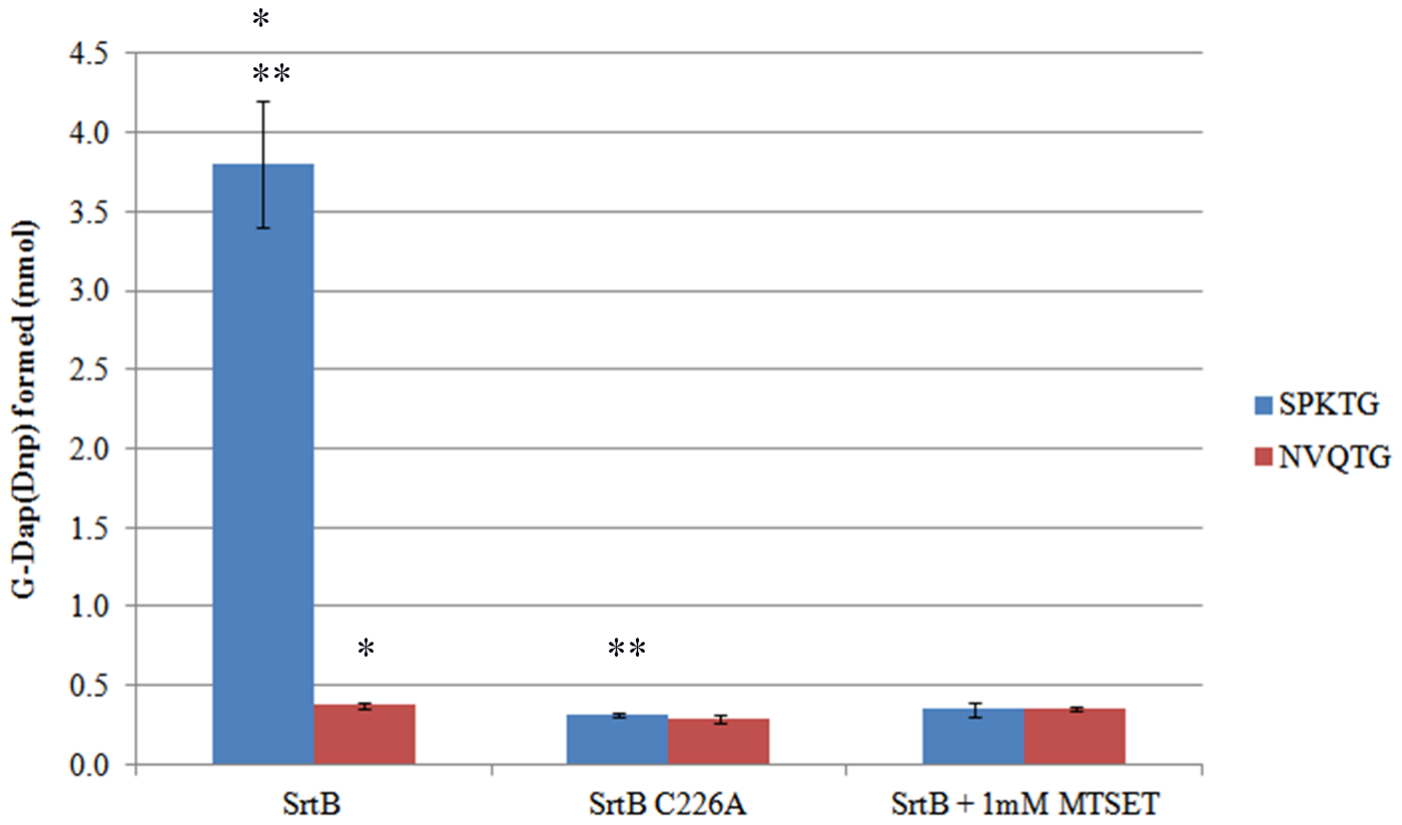
Cleavage of SPKTG and NVQTG Peptides by SrtB. Mean values (n = 3) are plotted with error bars representing +/− 1SD. Asterisks (*) and (**) indicate a p value of <0.01 as determined by Student's T-test.

**Figure 4:**
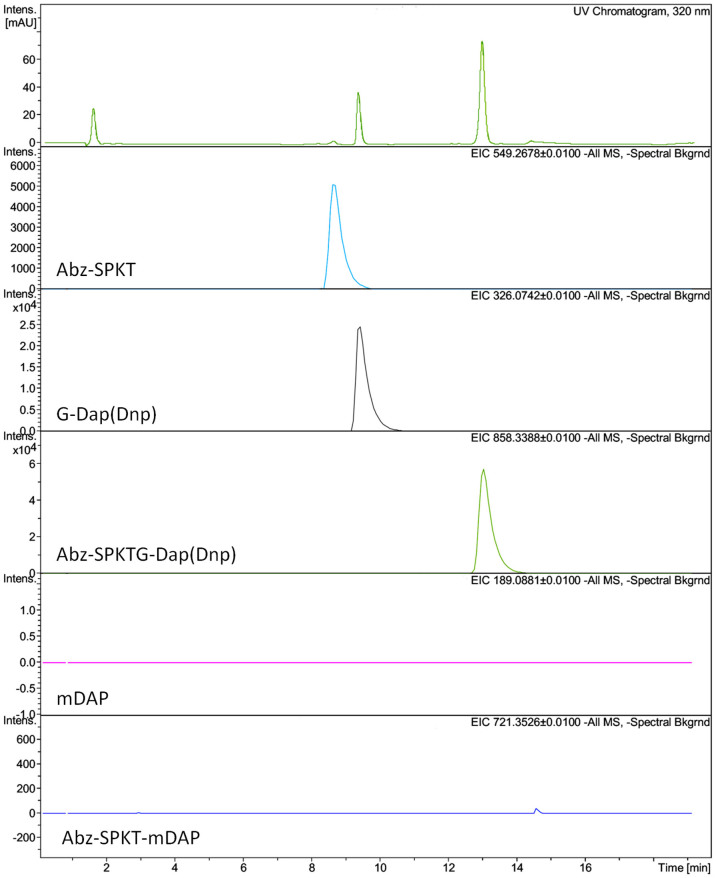
Extracted ion chromatographs for Abz-SPKTG-Dap(Dnp) cleavage by SrtB in absence of mDAP. The UV chromatograph is shown (top) followed by EICs for each of the analytes. The mass of each analyte is shown at the top right of the EIC and its putative assignment at the bottom left. Note that intensity scales are not equivalent between EICs.

**Figure 5:**
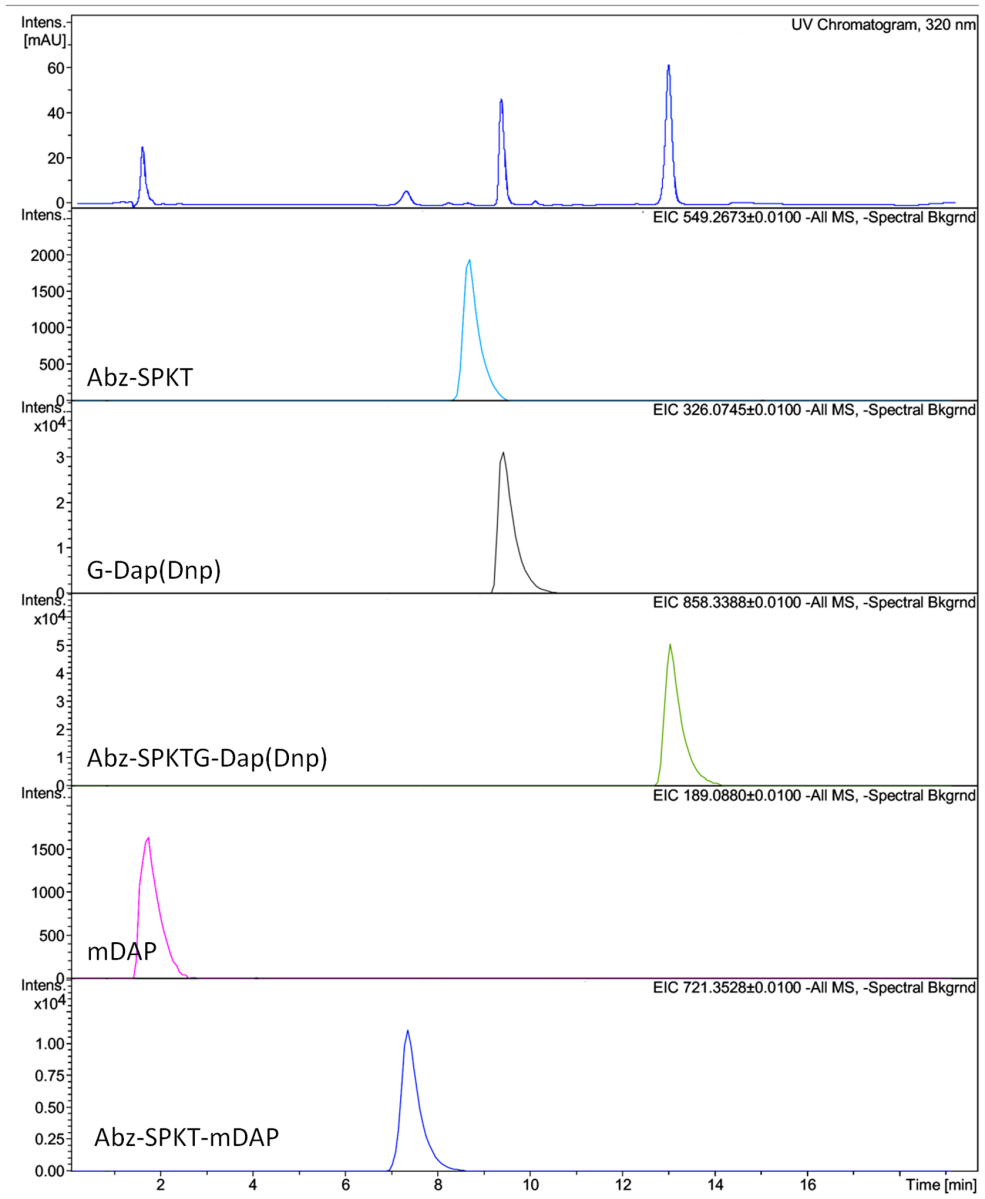
Extracted ion chromatographs for cleavage and transpeptidation of Abz-SPKTG-Dap(Dnp) by SrtB in the presence of mDAP. The UV chromatograph is shown (top) followed by EICs for each of the analytes. The mass of each analyte is shown at the top right of the EIC and its putative assignment at the bottom left. Note that intensity scales are not equivalent between EICs.

When incubated with recombinant *C. difficile* SrtB, the peptide Abz-SPKTG-Dap(Dnp) (a sortase substrate) is cleaved in two. No such cleavage occurs in the presence of SrtB C226A or SrtB + 1 mM MTSET, or when an NVQTG peptide is incubated with the active enzyme (Figure 3). Analysis by LC-MS confirms that cleavage of the peptide is at the threonyl glycyl bond (Figure 4). When the same reaction was performed in the presence of *meso*-DAP, an additional peptide is detected by mass spectroscopy, corresponding to the transpeptidation product Abz-SPKT-mDAP (Figure 5).

### Construction of a sortase deficient strain *C. difficile* 630 *srtB::erm*

**Figure 6:**
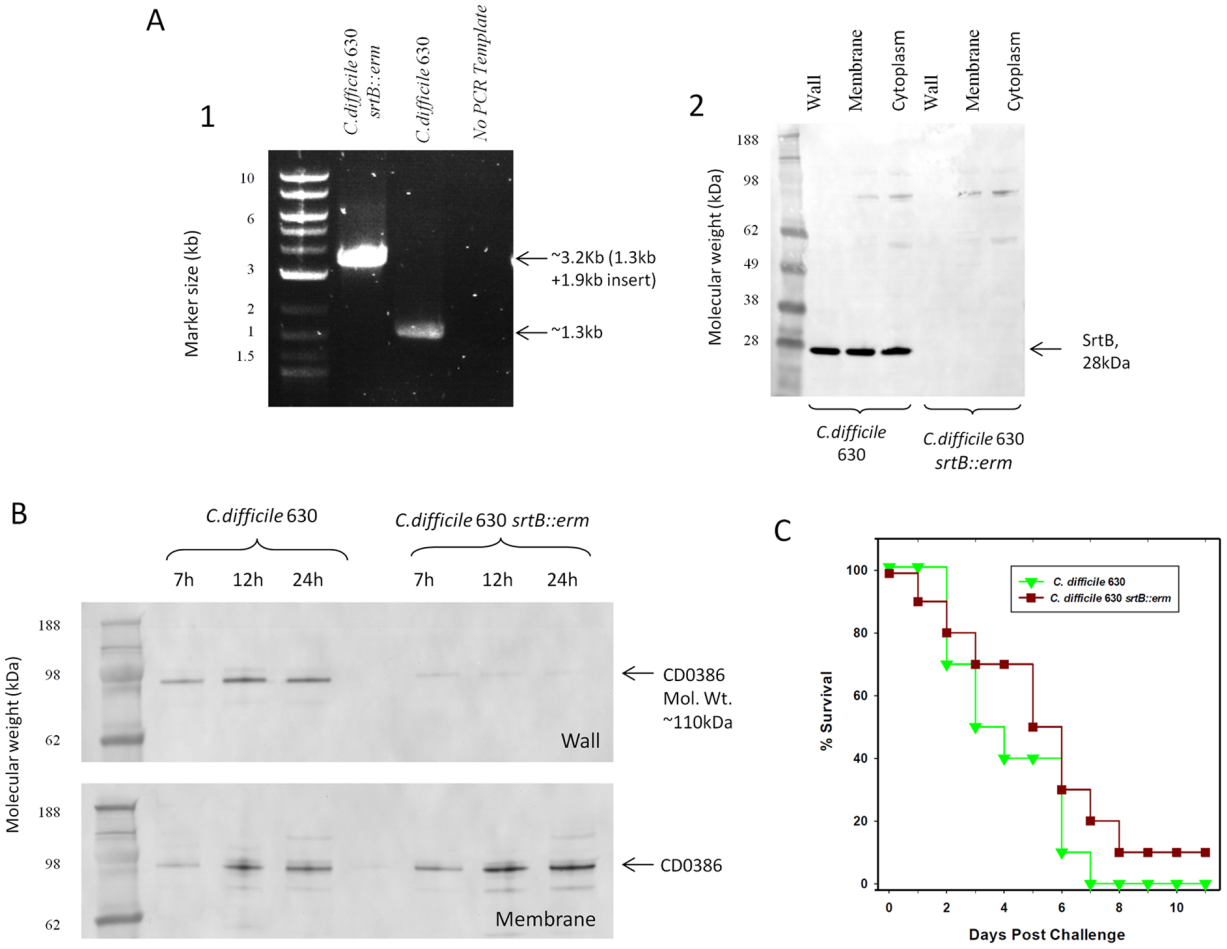
(A) Interruption of srtB by intron mutagenesis. (1) DNA fragments generated by PCR amplification utilising primers flanking the srtB gene, demonstrating an insertion of ~1.9 kb in strain 630 *srtB::erm* corresponding to the size of the Ll.LtrB intron. (2) Western blotting of cell fractions from 630 and 630 *srtB::erm* with antibodies raised against recombinant SrtB. (B) Cell fractionation and localisation of CD0386. Membrane and Wall fractions from *C. difficile* 630 and 630 *srtB::erm* after 7, 12 or 24 hrs of growth were analysed by SDS-PAGE, transferred to a nitrocellulose membrane and blotted with anti-CD0386 serum. Normalisation was effected by determining the protein concentration of each sample and loading an equal mass of protein from wild type and mutant fractions. The major bands in the figure correspond to CD0386. Note- A loss of CD0386 from the membrane fraction is observed. (C) Kaplan Meier plot of hamster challenge with *C. difficile* 630 and *C. difficile* 630 *srtB::erm*. Plotted points indicate the day on which hamsters were euthanized due to level 3 symptomatic scoring. No significant difference was observed, indicating that the sortase enzyme is not required for disease in the hamster model.

The retargeted intron was successfully introduced into *srtB*, resulting in a 1.9 kbp increase in PCR product length when the *srtB* gene was amplified by with flanking primers (Figure 6A1). Correct insertion of the intron was confirmed by sequencing of the *srtB* gene, and absence of SrtB confirmed by Western blotting (Figure 6A2).

### Localisation of putative sortase substrate CD0386 in *C. difficile* 630 *srtB::erm*

The wall localisation of CD0386, one of the putative *C. difficile* sortase substrates, was examined using antibodies raised against recombinant CD0386. Examination of wall and membrane fractions (Figure 6B) reveal that although CD0386 is present in the membrane fraction of both strains, there a significant loss of CD0386 from the membrane fraction of the sortase deficient strain *C. difficile* 630 *srtB::erm*.

### Challenge of hamsters with *C. difficile* 630 *srtB::erm*

A Kaplan-Meier plot of survival after challenge of Syrian hamsters with *C. difficile* 630Δerm and *C. difficile* 630 *srtB::erm* is shown in Figure 6C. In a similar experiment, survival of *C. difficile* 630 *srtB::erm* infected animal was also compared with *C. difficile* 630 wild type strain. In both instances, analysis of survival curves by Log-Rank test did not find any statistically significant difference between the mutant and wild type groups (p values = 0.24 and 0.51).

## Discussion

The *C. difficile* 630 genome possesses two open reading frames encoding proteins with homology to sortase enzymes, CD2718 (*srtB*) and CD3146. The latter of these is a pseudogene, containing an in-frame stop codon. Furthermore, this stop codon occurs upstream of the nucleotide sequence encoding the TLxTC active site motif, and the essential conserved arginine (analogous to *S. aureus* SrtA Arg197) is also absent, meaning that any expressed fragment is very unlikely to be active. SrtB possesses 37% amino acid sequence identity and 63% sequence similarity to the *B. anthracis* SrtB.

Since sortase enzymes have no function other than to anchor other proteins to the cell surface, the phenotype of a sortase mutant is determined entirely by the functions of the cell wall anchored proteins. The first aim of investigating a sortase mutant must, therefore, be to determine which surface proteins are anchored to the cell surface by the sortase and to confirm that their anchoring is absent in the sortase deficient strain. Following this, the wider phenotype of the sortase deficient organism can be investigated with the knowledge of the functions of its substrate proteins. Based upon analysis of the *C. difficile* genome using consensus searches designed to identify wall-sorted proteins, a *C. difficile* cell wall-sorting signal of SPxTG or PPxTG was initially suggested^21^. A similar methodology employed within a larger scale search of bacterial genomes identified seven putative substrates in *C. difficile*, six of which contained SPxTG motif and one with a PPxTG motif^22^. Following the methods of Comfort and Clubb^22^, we identified 7 genes encoding putative sortase substrates within the *C. difficile* 630 genome, shown in Table 1. In light of the classification of the *C. difficile* sortase as a class B enzyme, it is noteworthy that the proteins identified as its putative substrates are not typical of the repertoire observed for this class in other species. Notably absent are typical iron-associated class B sortase associated proteins such as haem binding proteins reflecting the role of SrtB in iron homeostasis in *S. aureus* and *B. anthracis*^23^,^24^. Based upon the list of proteins produced by our analysis, it would appear that although the *C. difficile* sortase is of the class B, it may fulfil a non-specialised role, more akin to the class A sortase of *S. aureus*.

Proteins CD0183 and CD2768 are putatively identified as peptidoglycan hydrolases. Such enzymes are essential for processes such as cell growth, remodelling and turnover of peptidoglycan and more specialised roles such as creation of space for secretion systems^25^. Localisation of peptidoglycan hydrolases to the cell surface is a common adaptation, presumably in order to allow efficient access to substrates - for example, the peptidoglycan hydrolase InlB of *Listeria monocytogenes* contains glycine tryptophan rich repeat regions which mediate interaction with the lipoteichoic acid of the cell wall^26^. Sortase anchoring of peptidoglycan hydrolases would simply represent another method of localisation. Protein CD2753 is putatively identified as a 5′-nucleotidase. While dephosphorylation of nucleotides by this protein may simply form part of a nucleotide scavenging pathway, there is evidence in other species that surface-localised nucleotidases may be virulence factors in their own right. For example, the AdsA protein of *S. aureus* is a wall-sorted 5′-nucleotidase which produces adenosine by cleavage of the adenosine monophosphate found at elevated concentrations at the site of infection and is essential for virulence in a mouse sepsis model^27^. A similar wall-sorted nucleotidase has been investigated in *Streptococcus sanguis*^28^. With respect to virulence, the proteins of most interest are those which display homology to adhesins of other species, namely CD0386, CD2831 and CD3392. There is evidence from an *in vivo* model that these proteins are virulence associated - in a porcine ligated loop model, transcription of CD0386 is upregulated 1.9- and 2.5-fold at 8 hrs and 12 hrs respectively. CD3392 and CD2831 are upregulated 2.7-fold and 2.4-fold respectively, but only at 12 hrs^29^. CD2831 and CD3382 possess conserved domains identified as 'Cna B' domains. Cna is a two domain collagen binding protein from *S. aureus* - domain A possesses collagen binding activity while the B domain forms a stalk which projects the binding domain away from the cell surface. These proteins therefore only share homology with the structural domain of a collagen binding protein and their annotation as ‘collagen binding protein' may therefore be premature. Perhaps the most intriguing of all the predicted sortase substrates is CD3246. Sequence analysis by Phyre2^30^ predicted a very low level of secondary structure, resulting in a failure to produce any reliable secondary structure homology results. Similarly, a BLAST search results in no proteins with significant homology outside of low complexity regions at the C and N termini. In spite of the lack of homologous proteins, some clues to the function of CD3246 have been uncovered by workers investigating riboswitches. Lee and co-workers^31^ discovered a self-splicing ribozyme upstream from the start codon of the CD3246 open reading frame. By monitoring cleavage of radiolabelled transcript, they discovered that the riboswitch is regulated by the presence of cyclic-di-guanidine monophosphate (c-di-GMP). The importance of c-di-GMP in regulation of bacterial virulence has only recently been recognised, and encompasses twitching motility in *Pseudomonas aeruginosa*, flagellar motility in *Salmonella typhimurium* and *Vibrio cholerae* and biofilm formation in all of the aforementioned species as well as *Yersina pestis*^32^. In these examples, c-di-GMP modulates virulence via direct effects on effector proteins rather than by the translational regulation described for CD3246.

The crystal structure of *C. difficile* SrtB C226A reveals a fold that is characteristic of the class B sortases, consisting of an eight stranded discontinuous beta barrel, decorated on its surface by several alpha and 3_10_ helices. The *C. difficile* SrtB C226A structure superposed with the class B sortases of *S. aureus* (PDB 1NG5), *B. anthracis* (PDB 1RZ2) and *Streptococcus pyogenes* (PDB 3PSQ). C^α^ root mean square deviation (RMSD) from *C. difficile* SrtB C226A with these structures were 1.41 Å, 1.19 Å and 1.62 Å respectively (Figure 1D).

Despite extensive attempts, it was not possible to crystallise wild type *C. difficile* SrtB, either under the optimal condition for SrtB C226A or any other condition tested in high throughput screening. A modelled active site was therefore constructed by reversing the mutation *in-silico* and adjusting the side chain torsion angles to match those of the analogous residues in the *S. aureus* and *B. anthracis* SrtB enzymes. The model assumes that the mutation did not lead to any gross changes in the structure of the enzyme – this assumption is supported by crystal structures of *S. aureus* SrtA, where SrtA (PDB 1T2P) and SrtA C186A (PDB 1T2O) structures can be superposed with an RMSD of only 0.6 Å. The modelled active site of *C. difficile* SrtB (Figure 2A) closely resembles those of other sortase enzymes, consisting of a catalytic cysteine flanked by arginine and histidine residues at distances of 6.2 Å and 5.8 Å respectively.

Attempts were made to co-crystallise SrtB C226A with an ‘SPKTG' peptide in order to provide insight into how the enzyme recognises this peptide substrate. These attempts proved unsuccessful, but in the absence of a structure, insights can be gained by analogy with enzyme-substrate complexes from other species. NMR structures of *S. aureus* SrtB covalently modified with a substrate mimetic peptide^33^ show the peptide sorting motif in a groove near the active site, with a floor provided by residues within strands β4 and β5 and whose walls are formed by residues projecting from loops connecting strands β6 to β7 (the β6/β7 loop), β7 to β8 (the β7/β8 loop), and β2 to β3 (the β2/β3 loop). The peptide is observed in an L-shaped structure, with the ‘NP' positions of the motif interacting substantially with the β6/β7 loop, particularly hydrophobic interactions from Tyr181 and Ile182, with additional hydrophobic contacts from Tyr128 and Leu96 and hydrogen bonds with Thr177, Asn92, Glu224 and Arg 233. As shown in Figure 2B, the β6/β7 loop of the *C. difficile* SrtB C226A contains an identical pairing of tyrosine and leucine, suggesting that the ‘SP' portion of the *C. difficile* sorting signal may be recognised in a similar manner.

As is observed in other class B sortases, the N-terminal region of *C. difficile* SrtB C226A forms two helices (H1 and H2) arranged in a ‘V' shape pointing away from the active site side of the enzyme. These features are unique to the class B sortases, and although their function has yet to be investigated, it has been suggested that they project the enzyme away from the cell wall. Until more is known regarding the function of these features, it is not possible to suggest the biological relevance of differences in this region.

Of the two peptides tested in a HPLC sortase assay, only the SPKTG peptide was cleaved by SrtB, confirming the findings of Donahue *et al*.^34^ that SPKTG is the cognate sorting motif of *C. difficile* SrtB. Furthermore, the lack of activity of SrtB against an NVQTG peptide casts doubt upon the suggestion by Tulli *et al*.^11^ that the collagen binding protein, CbpA, possessing an NVQTG motif, is sortase-anchored. Addition of the methanethiosulphonate reagent MTSET, or mutation of cysteine 226 to alanine both abolished all SrtB activity, confirming that this residue is essential for activity. MTSET reacts rapidly and specifically with the sulphydrl groups of cysteine residues, producing a disulphide-linked adduct. With the active site cysteine of a sortase enzyme modified by such a reaction, it is unable to participate in nucleophilic attack upon its peptide substrate and the enzyme is thus rendered inactive.

Even for the active SrtB enzyme, pilot kinetic analysis failed to observe meaningful rates of reaction, with an estimated maximal velocity of less than 0.5 nmol.hr^−1^. A complete kinetic characterisation was therefore not attempted. Low or undetectable activity in recombinant sortases has been observed previously^35^, in contrast to observations of rapid *in vivo* anchoring of sortase substrates in pulse-chase experiments^36^. Failure to detect significant SrtB activity may be due to a number of factors. Firstly, the short peptide substrates typically employed in sortase assays are poor surrogates of the enzyme's *in vivo* ligands, which are part of much larger proteins with significant secondary and tertiary structure. Secondly, such assays are generally performed in solution, whereas *in vivo*, the wall anchoring is a process intimately associated with the cell membrane increases the effective concentration of both substrates.

Characterisation the products of the reaction of SrtB with an SPKTG peptide in the presence or absence of *meso*-DAP clearly demonstrates that the *C. difficile* sortase is capable of the transpeptidation reactions characteristic of the sortase class, and responsible for their anchoring of proteins to the cell wall. It is notable that even when *meso*-DAP was in excess to the peptide substrates, the hydrolysis products were still observed. This is possibly an artefact of the *in vitro* reaction, as such *in vivo* would be highly unfavourable, leading to loss of the substrate. In the tightly surface localised environment in which sortase enzymes operate, the effective concentration of *meso*-DAP is likely to be much higher than was achieved in these experiments, leading to the transpeptidation reaction being favoured *in vivo*.

To determine the effect of sortase deficiency on anchoring of proteins to the cell surface, antibodies against the putative sortase substrate CD0386 were used to probe Western blots of subcellular fractions of a sortase knock-out strain. This revealed a partial loss of CD0386 in the wall fraction of the sortase deficient strain, despite its persistence in the membrane fractions. Tulli *et al*.^11^ recently reported the discovery of several *in vitro* inhibitors of *C. difficile* SrtB, raising the possibility of a sortase inhibitor based selective therapeutic for *C. difficile* infection. However, in our experiments it has been demonstrated that interruption of the *srtB* gene does not result in any statistically significant difference in mortality in the hamster model of *C. difficile* diarrhoea, suggesting that SrtB is not essential for virulence. There are multiple hypotheses which could explain such a lack of effect. Firstly, the proteins anchored to the cell wall by the sortase may not be required for virulence in the hamster model. Secondly, the effector proteins may be required for virulence, but still be displayed on the surface in the absence of sortase activity. There is evidence in other species that this could be the case - in *B. anthracis*, the C-terminus of a sortase conferred functional surface localisation to the phage receptor GamR even in the absence of sortase activity, a phenomenon that the authors suggested was due to membrane interactions from the hydrophobic portion of the C-terminal sorting signal^37^. Contrarily, investigations in other species have demonstrated that while sortase-linked surface proteins may still be present in the absence of sortase activity, they may not be displayed in a functionally appropriate manner – for example, inactivation of the *Streptococcus gordonii* sortase A has little effect on the surface expression of agglutinins SspA and SspB but leads to a 97% reduction in agglutination activity^38^.

Following the demonstration in this work that a functional sortase system operates in *C. difficile* and that its sorting signal includes an [SP]PxTG motif, investigation of the individual wall-anchored proteins may reveal novel adhesion factors and present novel opportunities to interfere with virulence. Regardless, the results presented here indicate that as far as can be assessed from the hamster model, inhibition of the sortase enzyme itself is unlikely to act as an effective anti-infective for therapy of *C. difficile* infections.

## Methods

### Growth of bacterial strains

*Escherichia coli* strains were cultured in lysogeny broth (LB, 10 g/L tryptone, 5 g/L yeast extract, 5 g/L NaCl) or on LB agar (LB + 1% agar). For expression of recombinant proteins, *E. coli* BL21 was grown in Terrific Broth (TB, 24 g/L tryptone, 42 g/L yeast extract, 17 mM KH_2_PO_4_ and 72 mM K_2_HPO_4_). *C. difficile* liquid cultures were grown in supplemented brain heart infusion broth (sBHI), consisting 36 g/L brain heart infusion (Oxoid), 5 g yeast extract, 0.5 g/l L-cysteine hydrochloride. Solid cultures were grown on fastidious anaerobe agar (FAA, Oxoid) or *C. difficile* selective agar (E&O laboratories).

### Identification and analysis of putative *C. difficile* wall-anchored proteins

The genome of *C. difficile* 630 was searched for proteins containing an [SP]PxTG motif using the Basic Local Alignment Search Tool (BLAST) of the National Centre for Biotechnology Information^39^. The resulting list of [SP]PxTG-containing proteins was further refined by removal of proteins which did not contain N-terminal secretion signal as predicted by the SignalP 4.0 program^40^, or those in which the [SP]PxTG motif was not within 50 amino acids of the C- terminus. To ascribe putative functions to the products of the identified genes, conserved domains were identified using the NCBI Conserved Domain Search^41^.

### Cloning and expression of recombinant *C. difficile* SrtB, SrtB C226A mutant and CD0386

A gene encoding *C. difficile* 630 open reading frame CD2718 (*srtB*) omitting amino acids 1–32 was synthesised by Entelechon GmBH and cloned into plasmid vector pEXP1 (Invitrogen) to generate pEXP-*srtB* such that the protein was fused with a C-terminal hexahistidine tag. Plasmid pEXP1-*srtB*-C226A, encoding a mutant of SrtB with a cysteine to alanine substitution at position 226, was generated by site directed mutagenesis. Briefly, the entire plasmid was amplified by polymerase chain reaction (PCR) using HiFi polymerase (Roche diagnostics) using the oligonucleotide primers (SrtBC226AF - GTTACGCTGTCTACTGCTACTTACGAATTCG, SrtBC226AR - CGAATTCGTAAGTAGCAGTAGACAGCGTAAC) at an annealing temperature of 65°C. The codon substitution was confirmed by sequencing of the *srtB* gene. A gene encoding *C. difficile* 630 CD0386 was cloned into pTAC-MAT1 such that the protein was fused with an N-terminal hexahistidine tag. *E. coli* Bl21DE3 (Invitrogen) transformed with pEXP1 SrtB, pEXP1 SrtB C226A or pTAC-MAT1 CD0386 were grown to an optical density of 0.6 in Terrific Broth. IPTG was added to a final concentration of 1 mM and growth continued at 16°C for a further 16 hrs. Cells were harvested by centrifugation for 30 mins at 3,500 g and resuspended 10% w/v in 25 mM HEPES pH7.5, 500 mM NaCl, 10 mM Imidazole.

### Purification of recombinant *C. difficile* SrtB and SrtB C226A proteins

All chromatographic procedures were performed using an AKTA FPLC system (GE Healthcare). *E. coli* BL21 expressing recombinant SrtB and SrtB C226A were lysed by sonication on ice for 10 × 30 s intervals and cell debris removed by centrifugation for 30 mins at 40,000 g followed by filtration with a 0.22 μm cellulose filter. Clarified lysate was applied to a 20 ml column of Ni^2+^-charged chelating sepharose (GE Healthcare) at a rate of 2 ml/min. Following washing with Buffer A (25 mM HEPES pH 7.5, 500 mM NaCl, 10 mM Imidazole), the flow rate was reduced to 1 ml/min and a gradient from 100% Buffer A to 100% Buffer B was initiated over a period of 40 mins, during which time 5 ml fractions were collected. Selected fractions were analysed by electrophoresis in a 6–12% Bis-Tris SDS-PAGE gel (Life Technologies). Eluate fractions containing a ~28 kDa protein corresponding to SrtB/SrtB C226A were pooled and dialysed extensively into 25 mM HEPES pH 7.5, 1 M (NH_4_)_2_SO_4_. Dialysed fractions were applied to a 5 ml butyl sepharose column (GE Healthcare) at a rate of 1 ml/min, during which time 5 ml fractions were collected, containing SrtB/SrtB C226A. Elution of contaminants was effected by washing of the column with 25 mM HEPES pH 7.5. Following analysis by SDS-PAGE, purified SrtB was dialysed extensively into a storage buffer consisting of 25 mM HEPES, 150 mM NaCl, concentrated to a target concentration of 10 mg/ml using a centrifugal concentrator with a 10 kDa MWCO (Sartorius) and stored at −80°C.

### Purification of recombinant CD0386 and production of polyclonal antisera

Following nickel affinity purification as described for SrtB, recombinant CD0386 was dialysed into 20 mM Bis-Tris pH 5.5, 180 mM NaCl and applied at a rate of 1 ml/min to an equilibrated MonoQ 5/50 anion exchange column (GE Healthcare). The column was washed until absorbance at 280 nm returned to the baseline level, at which point a gradient was initiated to 260 mM NaCl over 30 CV. Fractions containing CD0386 were dialysed into a storage buffer consisting 25 mM HEPES, 150 mM NaCl. Production of rabbit antisera was performed by Covalab S.A.S. A 50 μg dose of either SrtB or CD0386 was mixed with Freund's incomplete adjuvant and injected subcutaneously into duplicate New Zealand White rabbits at 0, 21 and 42 days. A terminal bleed was performed on day 53, and sera were assayed by ELISA with recombinant CD0386.

### Protein analysis by SDS-PAGE and Western blotting

Electrophoresis was performed for 35 mins at 200 V in a 4–12% Bis-Tris gel using a running buffer consisting of 50 mM MES pH 7.3, 50 mM Tris Base, 0.1% SDS, 1 mM EDTA. Normalisation between samples derived from *C. difficile* 630 and 630 *srtB::erm* was achieved by measuring the protein concentration of fractions using a bicinchoninic acid protein assay kit (Thermo) and ensuring that equal masses of protein were loaded between each strain. Gels were stained for 30 mins with NuPAGE SimplyBlue and destained overnight in distilled water. For analysis by Western blotting, proteins were electrophoresed as above and blotted onto PVDF membranes of 0.45 μm pore size. Membrane was washed once in TBS-T (50 mM Tris pH 7.5, 150 mM NaCl, 1% Tween 20) and blocked by incubation at 4°C overnight in 50 ml of blocking buffer (TBS-T, 5% skimmed milk powder). Rabbit serum was diluted 1:10,000 in 50 ml blocking buffer and incubated at room temperature for 90 mins with rocking agitation. Following washing with TBS-T, an alkaline phosphatase conjugated anti-rabbit antibody was added to the membrane diluted 1:50,000 in 50 ml blocking buffer. The membrane was washed thrice for 5 mins in 50 ml TBS-T before addition of nitro-blue tetrazolium/5-bromo-4-chloro-3'-indolyphosphate (NBT/BCIP) reagent (Thermo). Development was halted after 10 mins by extensive washing with distilled water.

### Crystallisation and X-ray diffraction of *C. difficile* SrtB C226A mutant

Conditions for crystallisation of SrtB C226A were determined by high throughput screening using an Art Robbins Phoenix robot to dispense 100 nl drops of 10 mg/ml SrtB C226A into 100 nl sitting drops of six commercially available 96-well screens (Molecular Dimensions). Small needle-like clusters were observed in the condition 0.1 M Phosphate-Citrate pH 4.2, 40% PEG300. The crystals used for X-ray diffraction were obtained by microseeding of these needle-like SrtB C226A crystals into the condition 0.1 M Phosphate-Citrate pH 4.2, 32% PEG300. X-ray diffraction data were collected at beam line I04-1 of the Diamond Light Source (Didcot, Oxon), UK. Due to the 40% PEG300 concentration in the crystallisation conditions, no additional cryoprotectant was added prior to X-ray diffraction data collection. Crystals were mounted within a cryoloop directly prior to data collection and frozen to 100 K by placement within liquid nitrogen stream. Diffraction images were processed in the orthorhombic space group P2_1_2_1_2_1_ using the XIA2 pipeline^42^ at the Diamond Light Source and merged using the program SCALA, a component of the CCP4 software suite^43^ (Table 1).

### Solution and refinement of *C. difficile* SrtB C226A structure

Initial phases were obtained by molecular replacement with PHASER-MR^17^, the search model being a homology model of *C. difficile* SrtB C226A generated by SWISS-MODELLER based upon the *B. anthracis* SrtB (PDB 1RZ2)^18^. Model building and structure refinement were performed using REFMAC^18^, each round of refinement consisting of three cycles of restrained refinement (Table 2). An appropriate conformation of a modelled Cys266 residue was chosen by constructing a superposition of *C. difficile* SrtB C226A with the *S. aureus* and *B. anthracis* SrtB structures and adjusting the χ1 angles of the mutated cysteine to match the conformation of the template structures. All figures were generated using the molecular visualization system PyMOL (Schrödinger).

### Assay of SrtB activity and LC-MS analysis of SrtB reaction products

Activity of SrtB was determined with a discontinuous HPLC assay based upon the method of Kruger *et al*.^44^ Peptides Abz-SPKTG-Dap(Dnp) and Abz-NVQTG-Dap(Dnp) were incubated with SrtB or SrtB C226A in a buffer of 25 mM HEPES pH 7.5, 150 mM NaCl. Reactions were quenched by rapid mixing with an equal volume of 25 mM HCl. Where required, inhibition of SrtB activity was achieved by the addition of 1 mM 2-(trimethylammonium)ethyl methanethiosulfonate (MTSET). Transpeptidation reactions were performed as above but with the addition of *meso*-diaminopimelic acid (*meso*-DAP) at a concentration equimolar to the tagged peptide substrate. Reaction samples were analysed by HPLC using a 250 mm × 4.6 mm octadecyl (C16) reverse phase column of 3 μm pore size (Supelco). Following equilibration of the column with 20% ACN, 80% H_2_O, the sample was injected and a gradient to 32% ACN, 68% H_2_O was initiated over a period of 12 mins. Through the analysis, absorbance was measured at 320 nm and 370 nm. Consumption of Abz-SPKTG-(Dap)Dnp was measured by integration of its corresponding peak with reference to a standard curve prepared with Abz-SPKTG-(Dap)Dnp standards in the range of 0–40 nmol. Reversed phase chromatography was performed on a Dionex Ultimate 3000 system using a 5 mm × 2.1 mm octadecyl uHPLC column (ACE HPLC), at a flow rate of 1 ml/min and 30°C column temperature. Analytes were eluted from the column using a gradient from 2–40% acetonitrile, 0.1% formic acid over 19 min. Electrospray Ionisation Mass Spectroscopy (ESI-MS) analysis of the eluate was performed by using a Bruker Microtof Q in full scan mode (+/− 50–1500 m/z). In addition to mass spectroscopic detection, eluted analytes were detected by UV absorbance at 320 nm. Spectral analysis was performed using the Bruker Compass suite.

### Generation of a *C. difficile* srtB gene interruption mutant

Targeted interruption of the *C. difficile* *srtB* gene was achieved using the Clostron system^45^. Targets for insertion were identified using the Targetron tool (Sigma Aldrich). The chosen insertion site within srtB contained the sequence (CTTTCTGTTGAGAATACAAATATAAATTAT*CCAGTTGTACAATCT) whereby the site of insertion is indicated by an asterisk. Plasmid pMTL007, containing the retargeted intron, was synthesised by DNA 2.0. *E. coli* CA343 was transformed with pMTL007 and grown to an optical density of 0.6 at 600 nm. A volume of 1 ml culture was centrifuged and resuspended in 100 μL of a *C. difficile* 630Δ*erm* culture of the same optical density to achieve a 1:10 ratio. The mixture was spread on a 22 μm nitrocellulose filter paper on the centre of a facultative anaerobe agar (FAA) plate. After 3 days of anaerobic incubation at 37°C, the slurry was washed from the filter paper and spread onto *C. difficile* selective agar plates (E&O Laboratories) supplemented with 15 μg/ml thiamphenicol to select for transconjugants. Integrants were selected by restreaking of transconjugant colonies onto BHI agar suuplemented with 5 μg/ml erythromycin. Colony PCR was performed with primers flanking the group I intron (ErmRF -ACGCGTTATATTGATAAAAATAATAATAGTGGG, ErmRR - ACGCGTGCGACTCATAGAATTATTTCCTCCCG) to confirm that the erythromycin resistant phenotype was due to the splicing out of the group I intron within the ErrmRAM. To confirm specific integration into the *srtB* gene, PCR was performed with primers flanking the gene of interest (SrtBflankF – TTCACTCAAAACCTTCACTCC, SrtBFlank R TCGATTCCTATCACCAGCTC) and the product sequenced. Absence of SrtB due to interruption of the SrtB gene was confirmed by Western blotting of whole cell extracts with polyclonal rabbit antiserum raised against recombinant SrtB purified as described above.

### Localisation of putative wall anchored protein CD0386 in *C. difficile* 630 *srtB::erm*

*C. difficile* 630 and 630 *srtB::erm* cells were fractionated using a method adapted from that of Jonquieres *et al.*^25^ Briefly, cells were resuspended in an iso-osmotic buffer supplemented with muramidases (100 mM Tris pH 6.9, 10 mM MgCl_2_, 0.5 M Sucrose, 1 mg/ml lysozyme, 60 μg/ml mutanolysin). After incubation at 37°C for 2 hrs, centrifugation at 3,000 g resulted in a soluble fraction and a pellet comprising protoplasts. Following osmotic lysis of protoplasts by resuspension in a low osmolarity buffer (10 mM Tris pH 7.4, 100 mM NaCl, 10 mM MgCl_2_), further centrifugation at 10,000 g resulted in a soluble fraction and a membrane pellet which was resuspended in 1 ml of the low osmolarity buffer and sonicated to homogeneity.

### Challenge of hamsters with *C. difficile* 630 *srtB::erm*

Procedures were approved by a local ethical committee and carried out under a UK Home Office-approved Project Licence and in accordance with the UK Animals (Scientific Procedures) Act 1986. Spores were prepared by inoculation of 12 Fastidious Anaerobe Agar (FAA) plates with overnight growth of either *C. difficile* 630 or *C. difficile* 630 *srtB::erm* in 5 ml sBHI. After 14 days of anaerobic incubation at 37°C, colonial growth was scraped with a sterile swab and resuspended in 15 ml Dulbecco's Modified Eagle's Medium (DMEM). The resuspension was heat-shocked at 62°C for 40 mins, aliquoted and stored at −80°C. Female Syrian hamsters (80–100 g) were housed in pairs in isolator cages with filter lids to minimise contamination between groups. Clindamycin was administered to all groups at a dose of 2 mg in 0.2 ml sterile H_2_O 48–72 h prior to challenge. Group 1 (n = 10) was challenged orally with either 10^3^ *C. difficile* 630 wild type strain or *C. difficile* 630Δ*erm* spores and Group 2 (n = 10) was challenged with 10^3^ *C. difficile* 630 *srtB::erm* spores. A control Group 3 (n = 6) remained unchallenged. All animals were weighed daily and monitored six times per day throughout the experiment. Hamsters were scored (0–3) on diarrhoea, weight loss, lethargy, and tender abdomen. Hamsters exhibiting advanced symptoms of disease were euthanized humanely.

## Author Contributions

C.J.C. performed the majority of the experiments, analysed the data and wrote the manuscript. A.K.R. and C.C.S. performed and supervised the biological experiments and edited the manuscript. K.R.A. supervised the structural and biochemical study, analysed the data and edited the manuscript. All authors reviewed the manuscript.

## Figures and Tables

**Table 1:** The putative *C. difficile* sortase substrates. Identification of *C. difficile* proteins fulfilling the criteria for wall anchored proteins, containing a C-terminal [SP]PxTG motif and a N-terminal secretion signal

ORF Name	Mass (kDa)	Gene Annotation	Predicted Conserved Domains (CDs)	E-Value for CDs
CD0183	37.0	Cell Wall Hydrolase	Pfam 877 (Unknown Function)	5.66e-36
			Pfam08239 (Bacterial SH3 domain)	1.12e-05
			COG0791 (Cell Wall Hydrolase)	1.94e-37
CD0386	111.6	Putative collagen-binding surface protein	COG4932 (Predicted Membrane Protein)	1.14e-10
CD2537	68.2	Membrane-associated 5'-nucleotidase	CD07407 (N-terminal metallophosphatase domain)	1.97e-101
			Pfam02872 (5′ nucleotidase, C-terminus)	8.37e-59
			COG0737 (5′nucleotidase/2′,3′-cyclic phosphodiesterase)	5.92e-94
CD2768	24.9	Cell Wall Hydrolase	Pfam 877 (Unknown Function)	2.42e-35
			Pfam08239 (Bacterial SH3 domain)	7.2e-11
			COG0791 (Cell Wall Hydrolase)	5.11e-35
CD2831	107.7	Putative Adhesin	Pfam05738 (Cna B Domain)	2.33e-06
			CL05349 (Collagen Binding Domain)	3.79e-03
CD3246	79.9	Surface Protein	Pfam06346 (Phormin Homology Region)	5.39e-05
CD3392	111.6	Cell Surface Protein	Pfam05738 (Cna B Domain)	1.47e-03

**Table 2:** Summary of crystallographic data for the SrtB C226A mutant structure

Data collection and refinement statistics	SrtB C226A
Space group (orthorhombic)	P2_1_2_1_2_1_
Number of protein molecules per asymmetric unit	2
Cell dimensions	a = 38.25Å, b = 90.27Å, c = 134.74Å
Resolution range (Å)	29.37–2.55
*R*_symm_ (outer shell)	0.151 (0.506)
I/σI (outer shell)	11.8 (4.2)
Completeness (outer shell) %	98.8 (100.0)
Total no. of reflections	122,009
Unique no. of reflections	15,902
*R*_cryst_/*R*_free_	0.196/0.262
**Average B-factor (Å^2^)**	
Overall	30.0
Water molecules	26.1
Protein atoms	29.8
Ligand atoms	43.7
Clash score	2.94
**RMS deviation**	
Bond length (Å)	0.009
Bond angle (°)	1.24
**Ramachandran statistics**	
Favoured (%)	94.3
Additionally allowed (%)	5.7

***Note****- R*_cryst _ = Σ*_h_*|*F*_o _− *F*_c_|/Σ*_h_F*_o_, where *F*_o_ and *F*_c_ are the observed and calculated structure factor amplitudes of reflection *h*, respectively. *R*_free_ is as for *R*_cryst_ for a randomly selected 5.0% subset of reflections not used in refinement.

## Notes

**Accession codes:** The atomic coordinates and structure factors (code 4UX7) have been deposited in the Protein Data Bank (http://wwpdb.org).
